# Unusually High Occupation of Co 3d State in Magnetic Weyl Semimetal Co_3_Sn_2_S_2_

**DOI:** 10.1021/acsnano.4c13750

**Published:** 2025-02-25

**Authors:** Jieyi Liu, Yiheng Yang, Jianlei Shen, Defa Liu, Gohil Singh Thakur, Charles Guillemard, Alevtina Smekhova, Houke Chen, Deepnarayan Biswas, Manuel Valvidares, Enke Liu, Claudia Felser, Tien-Lin Lee, Thorsten Hesjedal, Yulin Chen, Gerrit van der Laan

**Affiliations:** † Diamond Light Source, Harwell Science and Innovation Campus, Didcot OX11 0DE, U.K.; ‡ Clarendon Laboratory, Department of Physics, University of Oxford, Oxford OX1 3PU, U.K.; § Key Laboratory of Magnetic Molecules and Magnetic Information Materials of Ministry of Education and Research Institute of Materials Science, Shanxi Normal University, Taiyuan 030000, China; ∥ School of Physics and Astronomy, Beijing Normal University, Beijing 100875, China; ⊥ Key Laboratory of Multiscale Spin Physics, Ministry of Education, Beijing 100875, China; # Max Planck Institute for Chemical Physics of Solids, Dresden 01187, Germany; ¶ Department of Chemical Sciences, Indian Institute of Science Education and Research (IISER), Berhampur, Odisha 760003, India; ∇ ALBA Synchrotron, Carrer de la Llum 2-26, Cerdanyola del Vallès, Barcelona 08290, Spain; ○ Helmholtz-Zentrum Berlin für Materialien und Energie, Albert-Einstein-Strasse 15, Berlin 12489, Germany; ⧫ Beijing National Laboratory for Condensed Matter Physics, Institute of Physics, Chinese Academy of Sciences, Beijing 100190, China

**Keywords:** topological material, magnetic Weyl semimetal, XMCD, valency, Co_3_Sn_2_Sn_2_

## Abstract

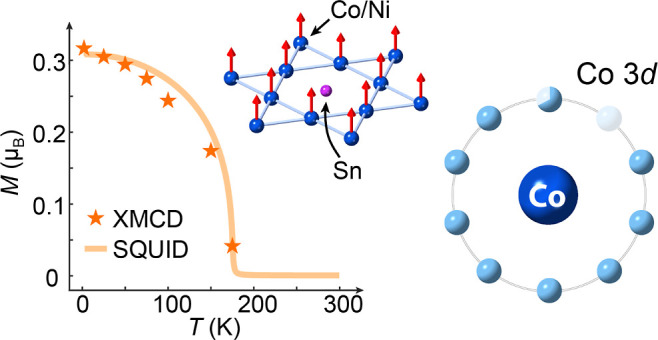

The physical properties of magnetic topological materials are strongly influenced by their nontrivial band topology coupled with the magnetic structure. Co_3_Sn_2_S_2_ is a ferromagnetic kagome Weyl semimetal displaying giant intrinsic anomalous Hall effect which can be further tuned via elemental doping, such as Ni substitution for Co. Despite significant interest, the exact valency of Co and the magnetic order of the Ni dopants remained unclear. Here, we report a study of Ni-doped Co_3_Sn_2_S_2_ single crystals using synchrotron-based X-ray magnetic circular dichroism (XMCD), X-ray photoelectron emission microscopy (XPEEM), and hard/soft X-ray photoemission spectroscopy (XPS) techniques. We confirm the presence of spin-dominated magnetism from Co in the host material, and also the establishment of ferromagnetic order from the Ni dopant. The oxygen-free photoemission spectrum of the Co 2p core levels in the crystal well resembles that of a metallic Co film, indicating a Co^0+^ valency. Surprisingly, we find the electron filling in the Co 3d state can reach 8.7–9.0 electrons in these single crystals. Our results highlight the importance of element-specific X-ray spectroscopy in understanding the electronic and magnetic properties that are fundamental to a heavily studied Weyl semimetal, which could aid in developing future spintronic applications based on magnetic topological materials.

## Introduction

Topological semimetals exhibit a low density of states near the Fermi level (*E*_F_), where the electronic structure is dominated by topologically nontrivial band crossings arising from bulk states.^1^ Examples of these crossing points near *E*_F_ include 4-fold-degenerate Dirac points,^2,3^ 2-fold-degenerate Weyl points,^4−6^ and nodal lines and nodal surfaces which consist of continuously connected Dirac or Weyl points in reciprocal space.^7^ Weyl semimetals (WSMs) are hosts of emergent bulk Weyl Fermions and surface Fermi arcs connecting Weyl nodes with opposite chirality. These unique features give rise to a range of exotic transport phenomena, such as negative magnetoresistance and the planar Hall effect, both of which are driven by the chiral anomaly.^8^ The WSM phase can occur in crystals that break either inversion symmetry or time-reversal symmetry, the latter often achieved through the introduction of magnetism.^9^ This symmetry breaking facilitates novel magnetic responses, making WSMs promising candidates for future spintronic applications.

**Figure 1:**
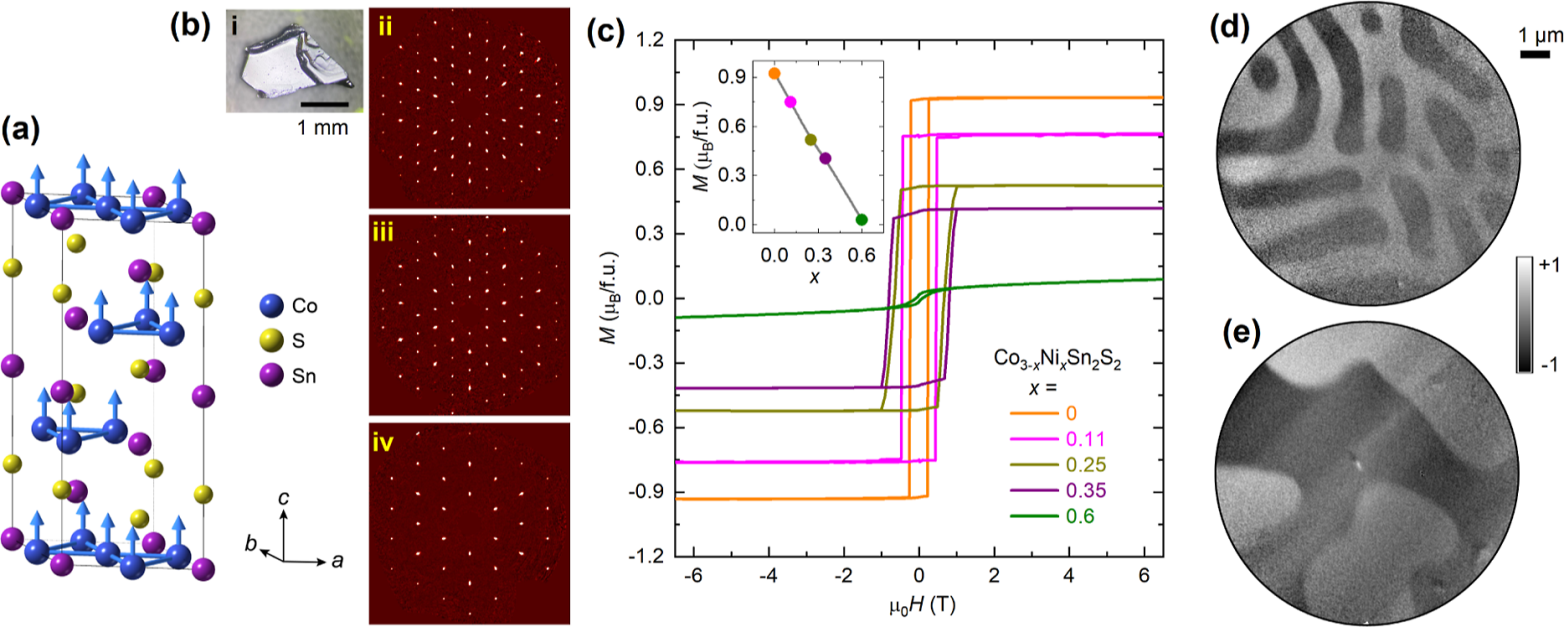
Crystal structure, bulk magnetic properties and magnetic domain images of Co_3–*x*_Ni_*x*_Sn_2_S_2_ single crystals. (a) Schematic of the crystal structure of Co_3_Sn_2_S_2_, with magnetic Co moments along the *c*-axis. [b(i)] Optical micrograph of a Co_2.65_Ni_0.35_Sn_2_S_2_ crystal and its X-ray diffraction patterns in the (ii) (0*kl*), (iii) (*k*0*l*), and (iv) (*hk*0) planes. (c) Bulk magnetization results of Ni doped crystals (*x* = 0, 0.11, 0.25, 0.35, and 0.6) with the field applied along the *c*-axis. Inset: Remanent magnetization as a function of doping concentration, showing a nearly linear behavior. (d) XPEEM image of a Co_2.65_Ni_0.35_Sn_2_S_2_ crystal taken at 37 K with the photon energy tuned to the Co L_3_ edge, showing magnetic domains after zero-field cooling. (e) XPEEM image collected at remanence under the same conditions after applying a small out-of-plane field pulse.

As one of the first experimentally confirmed magnetic WSMs,^10,11^ shandite Co_3_Sn_2_S_2_ crystallizes in a rhombohedral structure (space group *R*3̅*m*, no. 166) that exhibits quasi two-dimensional characteristics in the *ab*-plane, where Co_3_Sn layers are sandwiched between layers of S atoms, as shown in Figure 1a. This compound exhibits ferromagnetic order with a saturated moment of 0.3 μ_B_ per Co atom aligned along the *c*-axis, and a Curie temperature, *T*_C_, of 177 K.^12,13^ A giant intrinsic anomalous Hall effect has been discovered in pristine Co_3_Sn_2_S_2_, attributed to the large Berry curvature arising from its nontrivial topological bands.^14^ In these bands, Weyl points are located only 50 meV above the Fermi level, as revealed by angle-resolved photoemission spectroscopy experiments.^10^

Despite significant interest in the intimately intertwined electrical, electronic, and magnetic properties of Co_3_Sn_2_S_2_ over recent years, its magnetic structure remains a topic of active debate, particularly regarding the possible presence of a secondary antiferromagnetic or spin glass phase.^15−18^ Additionally, the contributions of spin and orbital moment to the total magnetic moment are not yet fully understood. While both the magnetic order and the robust topological surface states in Co_3_Sn_2_S_2_ are known to arise from Co 3d electrons, there are conflicting reports on the Co valence state, ranging from Co^0+^ to Co^2+^.^19−22^ Elemental doping further illustrates the correlation between electronic and transport properties in this WSM.^23−25^ Substituting Co with Ni in Co_3_Sn_2_S_2_ can enhance both the anomalous Hall conductivity and anomalous Hall angle by not only shifting the Weyl points closer to *E*_F_ through electron doping, but also by inducing local disorder that broadens the energy bands and therefore narrows the inverted gap.^26^ This results in an additional increase of the integrated Berry curvature. However, the exact electronic and magnetic configurations of Ni dopants have yet to be directly investigated.

Here, we report a comprehensive study of the element-specific magnetism and electronic configuration of Ni-doped Co_3_Sn_2_S_2_ single crystals using synchrotron-based X-ray techniques, including X-ray magnetic circular dichroism (XMCD), hard and soft X-ray photoemission spectroscopy (XPS), and X-ray photoelectron emission microscopy (XPEEM). Our results confirm the establishment of ferromagnetic order in both Co and Ni atoms, with Co magnetism primarily driven by spin moments, accompanied by a minor parallel contribution from orbital moments. Notably, we identify the unusual electron configuration in pristine Co_3_Sn_2_S_2_ as Co 3d^8.7^. Upon Ni doping, the filling of the 3d shell increases, reaching Co 3d^9.0^ in crystals with higher Ni concentrations. This unexpected increase in electron occupancy suggests a notable shift in the electronic structure with Ni substitution, providing new insights into the impact of elemental doping on the magnetic and electronic properties of Co_3_Sn_2_S_2_.

## Results

### Crystal Structure, Bulk Magnetic Properties and Magnetic Domains

We synthesized pristine and Ni-doped Co_3_Sn_2_S_2_ single crystals with varying Ni concentrations (*x* = 0, 0.11, 0.25, 0.35, and 0.6) in the form of Co_3–*x*_Ni_*x*_Sn_2_S_2_. The exact doping levels were verified using energy-dispersive X-ray spectroscopy and chemical analysis.^23,26^ In the crystals, Ni dopants randomly replace Co atoms within the kagome lattice, which retains the overall crystal structure.^27^ The structural stability is maintained since Ni_3_Sn_2_S_2_ at the highest Ni concentration shares the same lattice structure as Co_3_Sn_2_S_2_, and the atomic radii of Co and Ni are quite similar due to their proximity in the periodic table. Figure 1b(i) shows a photograph of a doped crystal with *x* = 0.35, exhibiting a flat, smooth *ab*-plane, characteristic of a quasi-2D kagome lattice. This *ab*-plane is the preferred cleavage plane, which was exploited for the X-ray spectroscopy studies. The high crystallinity of the sample is confirmed by the X-ray diffraction patterns shown in Figure 1b(ii–iv), which closely match the calculated patterns reported in ref (17). Figure 1c shows the bulk magnetization data measured at 2 K for all five doping levels, with the magnetic field applied along the *c*-axis of the samples. All crystals except for the highest doping level (*x* = 0.6) display square-shaped hysteresis loops, indicative of ferromagnetic order with a strong out-of-plane anisotropy. A monotonic decrease in magnetization is observed with increasing Ni concentration, which has been previously attributed to the increased spacing between the Co atoms upon Ni doping,^23^ which weakens the magnetic coupling between Co. Additionally, Ni may adopt a full 3d^10^ electronic configuration, as seen in Ni_3_Sn_2_S_2_, resulting in a nonmagnetic behavior.^28^ These observations suggest the need for further investigation into the possible magnetic order arising from Ni in the doped crystals.

The magnetic domain structure of the *x* = 0.35 crystal was imaged using XPEEM at the Co L_3_ edge at low temperature after zero-field cooling, as shown in Figure 1d. The magnetic Co domains exhibit striped or oval shapes, ∼μm in width. Upon applying a small out-of-plane magnetic field pulse (∼0.1 T), which is significantly smaller than the coercive field (∼0.8 T) required to fully align magnetic moments in this crystal, the number of domains within the field-of-view decreased, and the remaining domains expanded in size, shown in Figure 1e. This change confirms that the observed color contrast in the images is indeed of magnetic origin. While magnetic domains in Co_3_Sn_2_S_2_ have been previously studied using techniques such as magneto-optical Kerr effect microscopy,^29,30^ Lorentz microscopy,^31^ and magnetic force microscopy,^32^ to the best of our knowledge, this is the first synchrotron-based XPEEM study of Co_3_Sn_2_S_2_ single crystals. Our results demonstrate the technical feasibility of applying a high-voltage to cleaved single crystals, as required for XPEEM imaging, a method that offers unparalleled sub-10 nm spatial resolution while providing element-specific spectroscopic information.

### Spin and Orbital Magnetism in the Pristine Crystal

**Figure 2:**
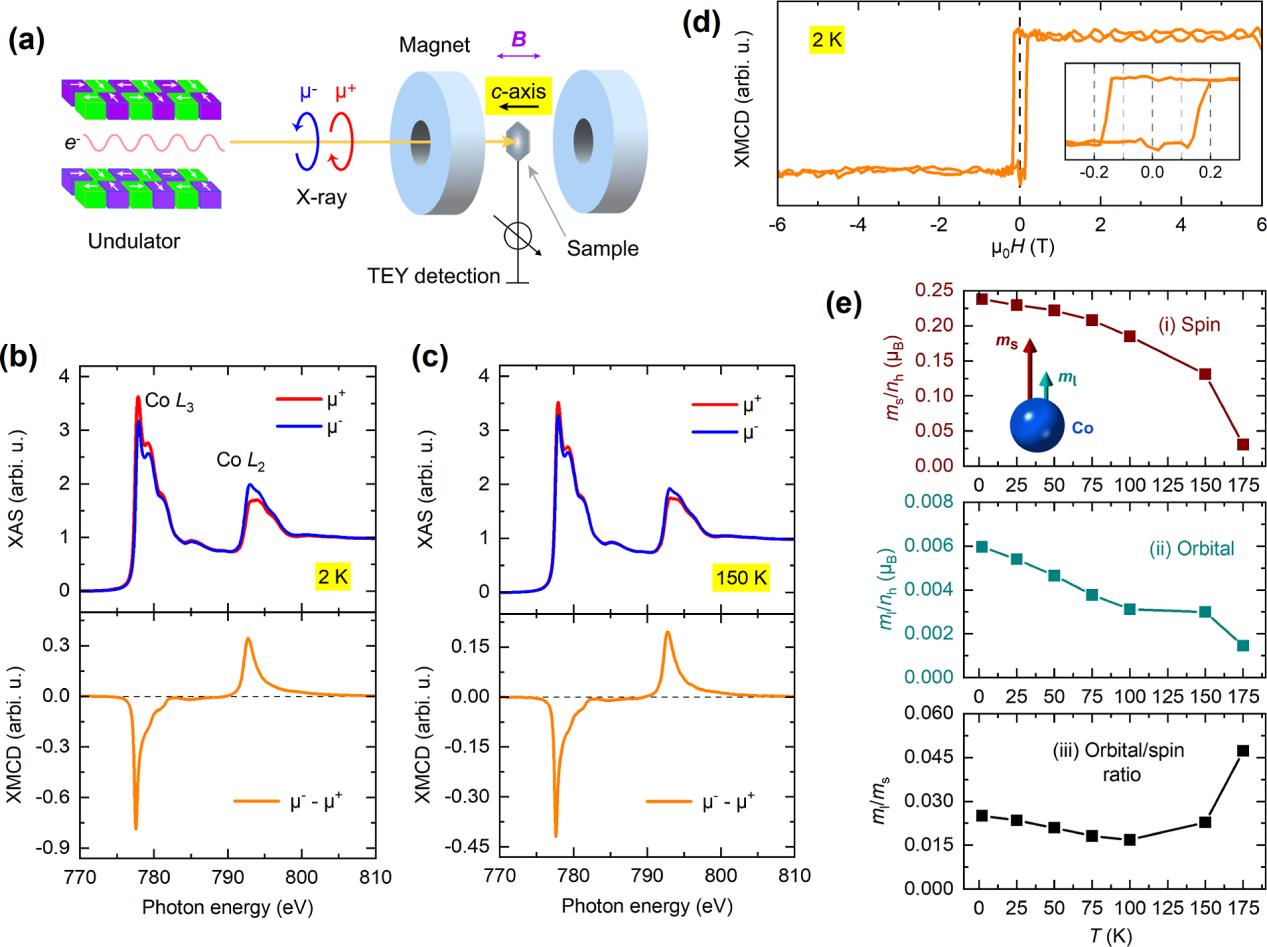
XMCD study of Co in a pristine Co_3_Sn_2_S_2_ crystal. (a) Schematic of the experimental setup for XMCD measurements with the magnetic field applied along the X-ray beam. The beamline optics between the undulator and endstation have been omitted for clarity. For these measurements, the field has been applied out-of-plane. XAS and XMCD spectra at the Co *L*_2,3_ edges measured in a field of 0.05 T at (b) 2 K and (c) 150 K. (d) XMCD hysteresis loop at 2 K, revealing out-of-plane magnetic anisotropy. The inset provides a detailed view of the hysteresis behavior at low magnetic fields. (e) Temperature dependence of the (i) spin moment and (ii) orbital moment per hole in the Co 3d shell, i.e., *m*_l_/*n*_h_ and *m*_s_/*n*_h_, along with (iii) the ratio of the orbital to spin moments. The inset in (i) illustrates a schematic representation of Co magnetization, highlighting a dominant spin contribution with a minor, positive orbital component.

We use XMCD to individually probe Co and Ni in Co_3–*x*_Ni_*x*_Sn_2_S_2_, to reveal the magnetic properties of these elements and to understand their contributions to the magnetic properties of the material. The experimental layout of the XMCD study is illustrated in Figure 2a, where both the X-ray beam and external magnetic field are aligned along the *c*-axis of the crystals to study the out-of-plane magnetization. Figure 2b shows X-ray absorption spectroscopy (XAS) data at the Co L_2,3_ edges using left- and right-circular polarization of the pristine Co_3_Sn_2_S_2_ crystal at the base temperature of 2 K in a small magnetic field of +0.05 T, after having applied a +6 T field to fully magnetize the sample. The line shapes are in good agreement with previous XAS results of Co_3_Sn_2_S_2_ reported in the literature.^22,33,34^ Here, we can see a clear difference in the spectra at the Co L_2,3_ edges using opposite circular polarizations, providing a sizable dichroism, also shown in Figure 2b. Pristine Co_3_Sn_2_S_2_ is known to be ferromagnetic with a Curie temperature *T*_C_ of 177 K. Therefore, close to *T*_C_, the net magnetic moment will be substantially reduced. As expected, at 150 K the material shows a smaller circular dichroism [Figure 2c], and both the XAS and XMCD line shapes closely resemble the low temperature ones. By tracking the XMCD at the Co L_3_ edge while sweeping the magnetic field, we obtain the Co hysteresis of the pristine crystal [Figure 2d], which shows a well-defined square-shaped loop with the saturation magnetization equivalent to the remanence value, confirming the ferromagnetic order of the Co atoms with the magnetization pointing along the *c*-axis. At the base temperature, we did not observe an exchange bias effect which was recently reported in a preprint using the same experimental procedure on Co_3_Sn_2_S_2_ crystals.^34^

**Figure 3:**
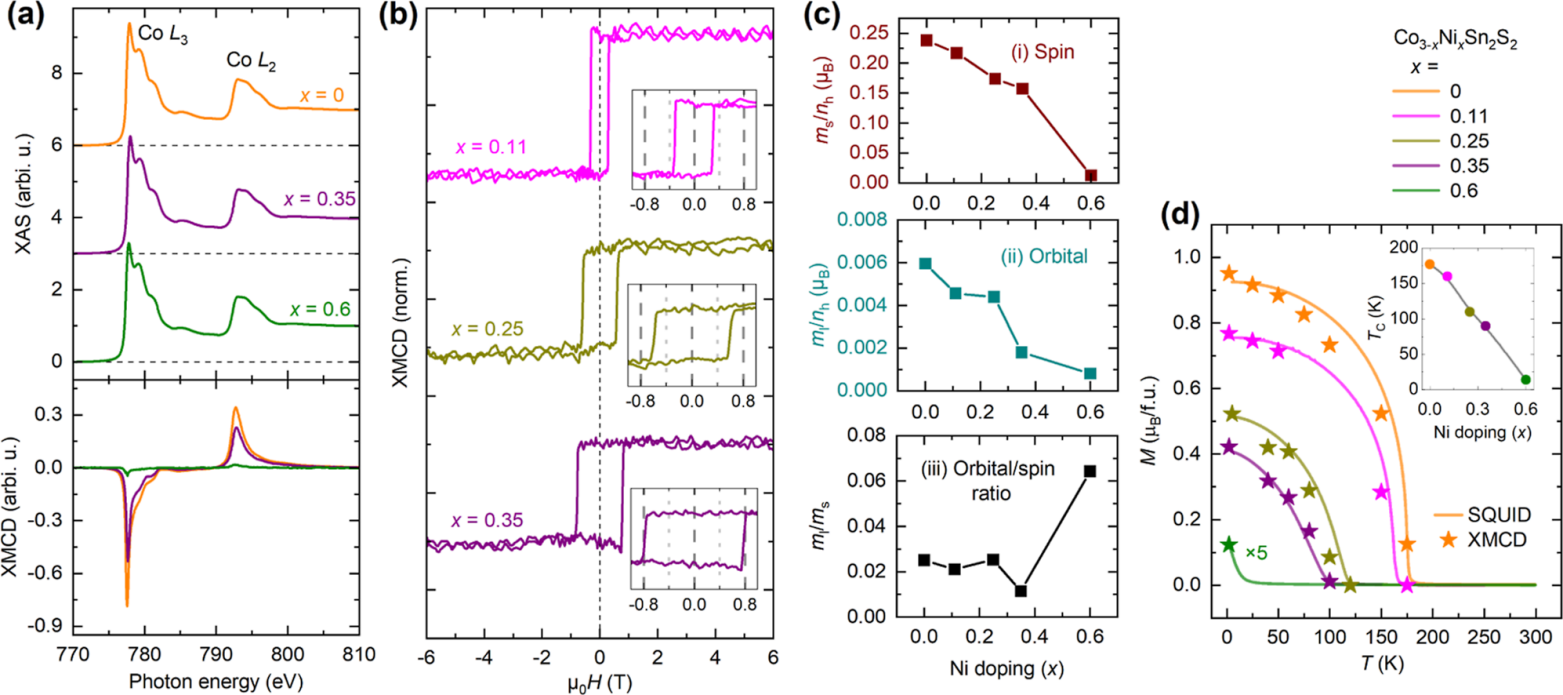
XMCD analysis of Co in Ni-doped Co_3–*x*_Ni_*x*_Sn_2_S_2_ crystals in out-of-plane magnetic fields. (a) XAS and XMCD spectra at the Co L_2,3_ edges measured at 2 K in a field of 0.05 T for Ni-doped crystals with doping levels *x* = 0, 0.35, and 0.6. The XAS spectra are vertically offset for better visualization. (b) XMCD hysteresis loops at 2 K for crystals with Ni doping levels of *x* = 0.11, 0.25, and 0.35. The insets provide magnified views of the hysteresis loops at low magnetic fields. (c) Dependence of the (i) spin moment and (ii) orbital moment per hole in the Co 3d shell on Ni doping at 2 K, i.e., *m*_l_/*n*_h_ and *m*_s_/*n*_h_, and (iii) the ratio of the orbital to spin moments, illustrating the impact of Ni substitution on magnetic behavior. (d) Temperature-dependent magnetization for all Co_3–*x*_Ni_*x*_Sn_2_S_2_ crystals with varying Ni doping concentrations, measured using SQUID (solid lines) and XMCD (individual data points, marked as stars), with *n*_h_ = 1.0–1.3 (see main text for detailed explanation). The inset shows the Curie temperature as a function of Ni doping concentration, revealing an approximately linear relationship.

Using the X-ray magneto-optical sum rules,^35,36^ we can deduce the spin and orbital moments of Co in Co_3_Sn_2_S_2_ at different temperatures. In the sum-rule analysis, both moments are proportional to the number of holes *n*_h_ in the Co 3d shell, which can contain up to 10 electrons when fully occupied. Without knowing the precise value of *n*_h_ in Co_3_Sn_2_S_2_, it is difficult to directly obtain the absolute values of orbital moment *m*_l_ and spin moment *m*_s_ per atom, however, we can confidently acquire the ratio between the two. Figure 2e displays the temperature dependence of the Co orbital and spin moments per hole, i.e., *m*_l_/*n*_h_ and *m*_s_/*n*_h_, and the ratio between them, i.e., *m*_l_/*m*_s_. As expected, the Co atoms possess a sizable spin moment which gradually deceases with increasing temperature, and vanishes at around 175 K, which agrees with our bulk magnetization data [see *x* = 0 data in Figure 3d], as well as with values reported in the literature.^14,37^ The orbital moment is aligned parallel to the spin moment, as expected from the Hund’s rule for more than half-filed shells, and also experiences a gradual decrease with its magnitude being ∼3% of that of the spin moment. This spin-dominated magnetism is common for 3d ferromagnetic systems. Our results agree with earlier studies by Yin et al.,^38^ who reported a small positive orbital moment on the order of 0.003 μ_B_ per Co atom contributed from all filled bands in Co_3_Sn_2_S_2_. Among these bands, their work highlighted the presence of a kagome flat band with a negative orbital moment of up to −3 μ_B_.

### Spin and Orbital Magnetism in the Ni Doped Crystals

Temperature-dependent XMCD measurements were also carried out on doped Co_3–*x*_Ni_*x*_Sn_2_S_2_ crystals, with Ni concentrations of *x* = 0.11, 0.25, 0.35, and 0.6. Figure 3a illustrates the averaged absorption spectra obtained with left- and right-circular polarization together with the resulting XMCD spectra at the Co L_2,3_ edges at 2 K. Here we only include the data for *x* = 0, 0.35, and 0.6 for presentation purposes, and apply a vertical baseline offset for better visibility. We do not find any noticeable differences in the shapes of the absorption spectra across the various doping concentrations. A clear monotonic decrease is observed in the XMCD magnitude at 2 K following the Ni content increase, consistent with the SQUID data. Similarly, element-specific hysteresis loops are produced by tracking the Co L_3_ XMCD while sweeping the magnetic field [Figure 3b]. Note that for the highest doped sample with *x* = 0.6, the dichroic signal is too small to record a hysteresis loop. Similar to pristine Co_3_Sn_2_S_2_, Co still imposes a strong out-of-plane anisotropy on the crystal at moderate Ni concentrations, as evidenced in the square-shaped hysteresis loops. The gradually enlarged coercivity has also been observed in SQUID and transport measurements, which may be explained by pinning of spins by Ni dopants in the kagome lattice.^23^ We also applied sum-rule analysis to all the crystals for which a sizable spin moment can be found along with a small, but positive orbital moment. The ratio *m*_l_/*m*_s_ is consistently below 8%, showing a universal spin-dominated magnetic response, as illustrated in Figure 3c.

Next, we performed a quantitative analysis and comparison between Co XMCD and bulk SQUID results across all doping concentrations. This comparison aims to address the ongoing debate regarding the valency of Co in Co_3_Sn_2_S_2_, as the sum-rule calculations used to extract the total spin and orbital moments depend on the number of holes *n*_h_ in the Co 3d shell. While Ni dopants also contribute to the overall ferromagnetic behavior, focusing on Co in this comparison is valid since the concentration of Ni is relatively small compared to Co, as we will demonstrate in the following subsections. Surprisingly, we found that the best agreement between the two measurement techniques, XMCD and SQUID, is achieved by assuming *n*_h_ = 1.0–1.3, depending on the doping concentration. This corresponds to a 3d electron count of 8.7–9.0, out of 10 in the full shell. Figure 3d shows the temperature-dependent magnetization probed by SQUID (solid lines) and XMCD (individual data points, marked as stars), showing a consistent trend across the different doping levels. In the “best-fit” scenario presented in this figure, the specific *n*_h_ values in Co_3–*x*_Ni_*x*_Sn_2_S_2_ are as follows
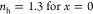

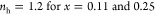

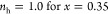


For the highest Ni concentration (*x* = 0.6), we also used *n*_h_ = 1.0, but choose to exclude this concentration from further discussion due to its small net magnetic moment, low Curie temperature, and lack of temperature-dependent XMCD data, which could lead to significant errors in quantitative comparison. The relatively small *n*_h_ values across all crystals are unusual, as Co ions in such compounds are typically treated as Co^2+^ (or Co^3+^), in which case the electronic configuration is [Ar] 3d^7^ (or 3d^6^). The inset of Figure 3d depicts an almost linear relationship between Ni concentration and Curie temperature, as derived from SQUID measurements, which is also in quantitative agreement with the XMCD data.

### Elemental Valency Probed by X-ray Photoemission Spectroscopy

To fully understand the valency and electron configuration of each element in Co_3–*x*_Ni_*x*_Sn_2_S_2_ crystals, we carried out synchrotron-based XPS studies using both soft X-rays (SX) at 1300–1500 eV for surface-sensitive measurements and hard X-rays (HX) at 6600 eV for bulk-sensitive measurements, all aligned onto the same sample spot. The crystals were measured at 90 K after cleaved in situ under ultrahigh vacuum to prevent oxidation, a common issue in lab-based XPS. We also repeated the same measurements at 30 K and observed no significant differences between the two temperatures, nor did we find any variation in the data when probing different regions of the crystal. As a reference, a 7.4 nm-thick Co film was grown in situ on an Au(111) substrate using e-beam evaporation.^39^ This reference was used to validate the Co valency and to compare surface and bulk characteristics in the Co_3–*x*_Ni_*x*_Sn_2_S_2_ samples.

**Figure 4:**
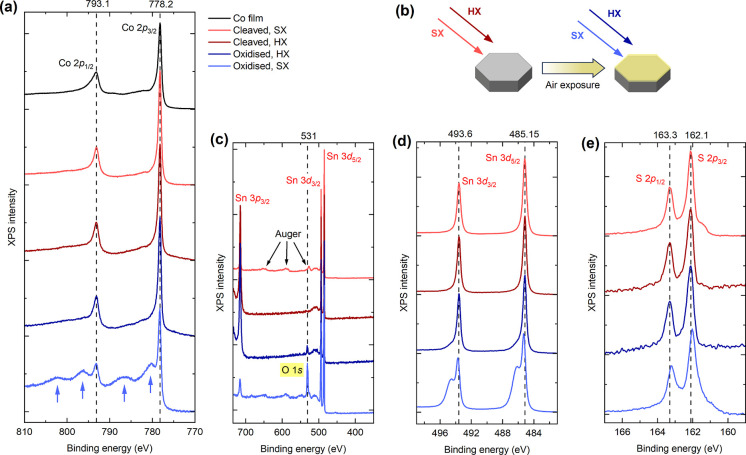
Soft and hard XPS study of Co_2.65_Ni_0.35_Sn_2_S_2_ crystals. (a) XPS spectra of the Co 2p_1/2_ and 2p_3/2_ core levels obtained using soft X-ray (SX, 1300–1500 eV) and hard X-ray (HX, 6600 eV) sources. The spectra were measured after fresh cleavage (in light and dark red) and after deliberate air exposure (in light and dark blue). Additional peaks (marked by light blue arrows) emerge after air exposure, indicating surface oxidation. A reference spectrum from a metallic Co film (in black) is included for comparison. (b) Schematic representation of the experimental procedure used for air exposure to induce oxidation of the crystal surface. (c) XPS survey scan showing the appearance of the O 1s core level after air exposure, confirming surface oxidation. Detailed XPS spectra of (d) Sn 3d_3/2_ and 3d_5/2_ and (e) S 2p_1/2_ and 2p_3/2_ core levels.

Figure 4a shows the XPS spectra covering the Co 2p levels of the *x* = 0.35 crystal using SX (in light red) and HX (in dark red), along with the Co film using SX (in black). The base lines of the spectra are deliberately offset in the vertical direction for clarity. Clearly, the line shapes and peak positions in both energy ranges quantitatively agree with each other, indicating a good uniformity in Co valency across surface and bulk of the material. The 2p_1/2_ and 2p_3/2_ peaks are located at a binding energy of 778.1 and 793.0 eV, respectively, with a spin–orbit coupling induced separation of 14.9 eV. The Co 2p peak positions of the Co film overlap very well with those of the crystal, also showing peak maxima at 778.1 and 793.0 eV. In fact, there is hardly any visible multiplet structure in these spectra. An asymmetric line shape along with a small plasmonic energy loss structure can be observed for all three spectra, indicating that both the crystal and the film have good metallicity. The quantitative agreement between the peak positions and shapes of the Co 2p core levels in both the crystal and the reference Co film supports claims by several research groups that the Co valency in Co_3_Sn_2_S_2_ is close to Co^0+^,^20,33^ or in some cases expressed as Co^δ+^ indicating a small positive valency.^21,40^ However, the satellite peaks at higher binding energies near the Co 2p_1/2_ and 2p_3/2_ peaks, which have been reported in previous studies,^21,33,40^ are absent in our data.

To investigate the impact of oxidation on the XPS spectra, we removed the crystal from the ultrahigh vacuum chamber and deliberately exposed it to air for 10 min before repeating the measurements under identical experimental conditions, as illustrated in Figure 4b. The postoxidation Co 2p spectra, displayed at the bottom of Figure 4a, reveal four additional humps at binding energies 780.3, 786.3, 796.2, and 802.2 eV (marked by light blue arrows). These peaks observed under SX (in light blue), that closely match the features in photoemission spectrum of CoO,^41^ are characteristic of surface oxidation and resemble the satellite structure previously reported in Co_3_Sn_2_S_2_.^21,33,40^ In contrast, our data obtained from HX (dark blue) remained largely unchanged after oxidation, further confirming that these extra peaks result from surface oxidation. Throughout the process, we also monitored the O 1s peak at 543 eV in the survey scans, as shown in Figure 4c. In freshly cleaved samples, no oxygen signal was detected in either SX or HX scans. However, after air exposure, the O 1s peak became prominent in the SX survey scan, while it appeared weaker in the HX scan due to the bulk sensitivity of HX. Furthermore, a C 1s peak at 248.7 eV was observed (not shown in the Figure) in the SX data following air exposure, but this feature was absent in the other three survey scans.

In addition to Co, we observed consistent surface- and bulk-sensitive spectra for Sn 3d and S 2p after fresh cleaving, as shown in Figure 4d,e. The Sn 3d_3/2_ and 3d_5/2_ peaks were located at binding energies of 493.6 and 485.15 eV, respectively, while the S 2p_1/2_ and 2p_3/2_ peaks appeared at 162.1 and 163.3 eV. A shoulder at 161.5 eV, next to the main S 2p_3/2_ peak, can be attributed to a surface contribution.^21,42^ Similar to Co, the bulk-sensitive HX spectra for both Sn 3d and S 2p remained unaffected by air exposure, while additional peaks at 486.1 and 494.6 eV emerged in the SX spectrum. These peaks, shifted by 1.0 eV above the Sn 3d peaks, clearly indicate surface oxidation. We repeated this on a crystal with *x* = 0.25 before and after air exposure, which produced spectra with a similar line shape and identical peak positions to those of the *x* = 0.35 crystal. Our results highlight the critical importance of in situ cleaving for probing the elemental valency of single crystals using XPS.

### Magnetism of the Ni Dopant

**Figure 5:**
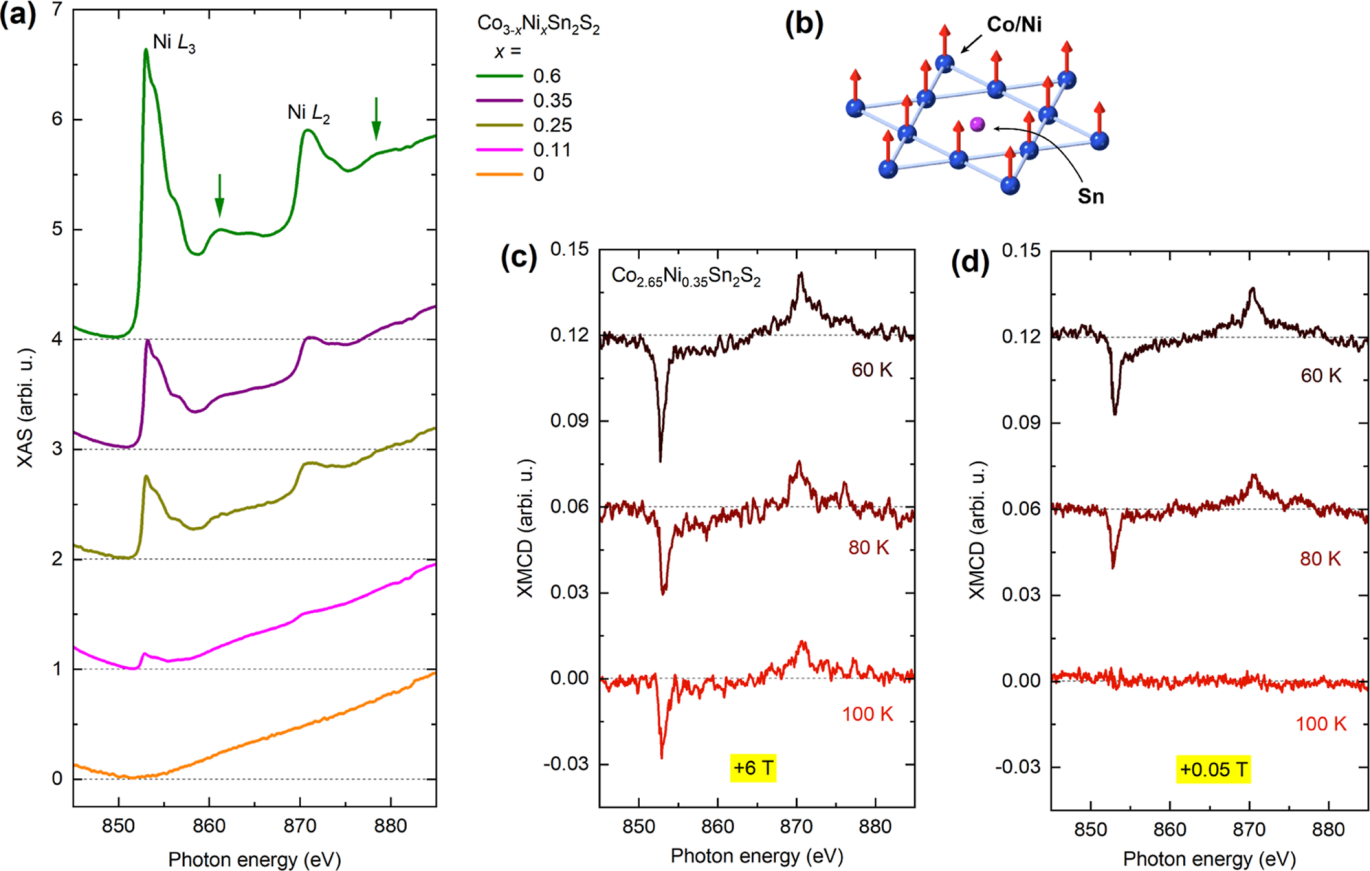
XMCD analysis of Ni in Ni-doped Co_3–*x*_Ni_*x*_Sn_2_S_2_ crystals. (a) XAS spectra at the Ni L_2,3_ edges measured at 2 K for Ni-doped crystals, including a pristine (undoped) crystal as a reference. The green arrows mark satellite peaks associated with Ni doping. The spectra are vertically offset for clarity. (b) Schematic illustration of the Co kagome plane with Ni substitution, highlighting the ferromagnetic order of both Co and Ni in the doped lattice. Temperature-dependent XMCD spectra at the Ni L_2,3_ for the Co_2.65_Ni_0.35_Sn_2_S_2_ crystal measured (c) in a magnetic field of +6 T and (d) in a small field of +0.05 T.

Following the detailed analysis of the properties of Co, we now turn to the valency and magnetism of the Ni dopants, as investigated using XMCD. The proximity of the Ni L_2,3_ edges (870 and 853 eV) to those of Co L_2,3_ leads to some overlaps in the absorption spectra due the extended fine structure of Co, and the difference in relative volumes of Co and Ni further complicates the analysis. Figure 5a shows the XAS spectra for all five crystals in the 845–885 eV range, averaged over left- and right-circular polarizations. The spectra are normalized using the pre-Ni L_3_ and post-Ni L_2_ slopes, with pristine Co_2_Sn_2_S_2_ (in orange) serving as a reference. As expected, the contribution from Ni becomes more dominant as the doping increases, progressively overshadowing the Co background. The Ni 3d^10^ configuration in the hybridized ground state is not visible in XAS, as the excited core electron cannot be accommodated in a full 3d shell. The main absorption peaks at 853 and 870 eV are characteristic of the Ni 3d^9^ configuration, while the two satellite peaks at 861 and 878 eV (marked by green arrows) correspond to the Ni 3d^8^ configuration.^43^ Notably, these satellite features are already observable in the *x* = 0.25 crystal and become increasingly pronounced with higher Ni concentrations. Combined with the fact that Ni in pristine Ni_3_Sn_2_S_2_ possess a full 3d^10^ shell,^28^ our result suggests electron charge transfer from Ni to other elements in the doped crystals.

To gain insight into potential magnetic order from Ni, we carried out temperature-dependent Ni L_2,3_ XMCD measurements on the *x* = 0.25 and 0.35 crystals. The medium Ni concentrations were chosen as they offer a balance between strong absorption from Ni and significant magnetization with a high *T*_C_. Here, we focus on the *x* = 0.35 crystal, particularly around the bulk magnetic transition near *T*_C_ = 90 K. Figure 5c,d show the Ni XMCD spectra at +6 T and +0.05 T, normalized to the same XAS magnitude and vertically offset for clarity. A clear net dichroism is observed at both +6 T and +0.05 T below 80 K, confirming that ferromagnetic order is established in Ni. At 100 K, although a sizable moment can still be induced by the 6 T field, the moment vanishes at remanence, indicating the loss of ferromagnetic order. This result confirms that Ni in the *x* = 0.35 crystal is ferromagnetic with a *T*_C_ between 80 and 100 K, aligning well with the *T*_C_ = 90 K obtained from bulk magnetometry. The correlation between the magnetic orders of Co and Ni within the kagome lattice suggests that the two elements share the same Curie temperature, likely due to their magnetic coupling.

Due to the significant background from the Co absorption edges, direct sum-rule analysis to quantify the magnetic moment of Ni is not feasible. However, we can estimate the Ni moment by comparing the magnitude ratio between XMCD and XAS at the Ni L_3_ edge. For reference, a ratio of 20% in pure Ni corresponds to a magnetic moment of 0.57 μ_B_,^44^ while a 50% ratio in Ni_13_^+^ clusters yields a magnetic moment of 1.46 μ_B_.^45^ In our case, as shown in Figure 5d, when the XAS at the Ni L_3_ edge for the *x* = 0.35 sample is normalized to 1, the corresponding XMCD at Ni L_3_ reaches 0.03 at 60 K under a field of 0.05 T, giving a ratio of 3%. This allows us to estimate the magnetic moment per Ni atom to be on the order of 0.09 μ_B_, resulting in only 0.03 μ_B_ for the Ni moments per formula unit in Co_2.65_Ni_0.35_Sn_2_S_2_. We can therefore neglect the contribution of Ni in bulk magnetization measurements of doped crystals when they are quantitatively compared with Co L_2,3_ XMCD results.

## Discussion

Before we further explore the valence state of Co_3_Sn_2_S_2_, it is useful to first consider Ni_3_Sn_2_S_2_, where Ni possesses one extra electron in its 3d shell compared to Co. Previous studies using ^61^Ni and ^119^Sn Mössbauer spectroscopy and XPS concluded that the Ni 3d band is completely filled with 10 electrons, giving a valence state of Ni^0+^.^28^ This conclusion was later supported by Aziz et al.,^46^ who confirmed the Ni 3d^10^ configuration using band structure calculations and Bader charge analysis. The full shell configuration is also reflected in the fact that pristine Ni_3_Sn_2_S_2_ does not exhibit magnetic order. However, as a prerequisite for establishing magnetic order, there must be holes in the Ni 3d shell of the doped crystals with *x* = 0.25 and 0.35. The clear Ni L_3_ white line in the XAS data, which is due to the intense absorption in the near edge region, further confirms that Ni does not possess a fully occupied 3d shell in any of the Co_3–*x*_Ni_*x*_Sn_2_S_2_ crystals we studied.

Ni_3_Sn_2_S_2_ and Co_3_Sn_2_S_2_ share the same crystal structure and potentially similar chemical bonding within the lattice, with the key difference being the electron filling of the Ni/Co 3d shell. It therefore seems plausible that the Co 3d shell would possess one less electron than Ni 3d^10^ in TM_3_Sn_2_S_2_ (TM = transition metal), resulting in a Co 3d^9^ configuration, in agreement with our sum-rule analysis. This analogy also helps to explain the sharp reduction in ferromagnetism as the Ni concentration increases in Co_3–*x*_Ni_*x*_Sn_2_S_2_. Even at *x* = 0.6, where the ratio of Co to Ni atoms is still 4:1, both the magnetization and Curie temperature approach zero. The presence of satellite peaks in the Ni L_2,3_ edge spectra that represent a Ni 3d^8^ contribution in medium to highly doped samples suggests a charge-transfer mechanism, where electrons are transferred from Ni 3d^10^ to Co 3d^8.7^ shell in the doped crystals. As a result, the Co 3d shell approaches full occupancy with increasing Ni substitution, thereby suppressing the ferromagnetic order.

As one of the first experimentally confirmed magnetic Weyl semimetals, Co_3_Sn_2_S_2_ is no stranger to experiments aiming at revealing the electron configuration, in particular using XPS.^19−21,33,40,42,47^ By comparison with our XPS data before and after air exposure, we can conclude that while some published results were obtained under oxygen-free conditions,^42^ the majority were affected by surface oxidation,^19,21,33,40,47^ potentially complicating interpretations of elemental valencies. Meanwhile, oxidation does not significantly shift the main Co 2p_3/2_ and 2p_1/2_ peak positions, which still coincide with those of pure Co. Overlap of these peak positions has been used by several groups to support the idea that Co in Co_3_Sn_2_S_2_ is close to 0 + valency.^21,33,40^

## Conclusion

We carried out comprehensive synchrotron-based X-ray spectroscopy experiments on ferromagnetic Weyl semimetal Co_3–*x*_Ni_*x*_Sn_2_S_2_ single crystals with varying Ni doping concentrations (*x* = 0, 0.11, 0.25, 0.35, and 0.6). Our controlled air exposure studies revealed oxygen-free core-level photoemission spectra of Co in the unexposed crystals, which closely resembles that of a pure Co film, suggesting a Co valency near 0. The doping-dependent XMCD study confirms that magnetism in Co is dominated by spin moments, with a small but positive orbital contribution. Additionally, the Ni dopants exhibit ferromagnetic behavior with a Curie temperature consistent with that of the host material. A significant finding of this study is the unusually high electron occupation in the Co 3d shell, reaching ∼8.7 in pristine Co_3_Sn_2_S_2_, and this occupation increases to 9.0 with Ni doping, likely due to a charge-transfer mechanism from Ni to Co. Modifications in the Co/Ni 3d shell may affect the vertical hybridization between the Co 3d and Sn 5p electrons, potentially reshaping the surface kagome electronic states in doped Co_3_Sn_2_S_2_.^48^ Furthermore, using core-level PEEM, we successfully imaged ferromagnetic domains of Co, characterized by oval and stripe shapes, with high spatial resolution. This result is a crucial step in addressing the discrepancies in the understanding of secondary magnetic phases in Co_3_Sn_2_S_2_. Our study highlights the importance of X-ray spectroscopy for a thorough comprehension of element-specific electronic and magnetic characteristics of Weyl semimetals. Our insights are vital for advancing the development of spintronic applications based on the unique physical properties of magnetic topological materials.

## Experimental Methods

### Sample Growth, Composition, Structure and Bulk Magnetization

Co_3–*x*_Ni_*x*_Sn_2_S_2_ single crystals were synthesized using the self-flux method for Ni doping concentrations of *x* = 0.25, 0.35, and 0.6, and mixed flux method of Sn and Pb for *x* = 0 and 0.11. The exact stoichiometry of the synthesized crystals was verified through energy dispersive X-ray spectroscopy and chemical analysis, as previously reported.^23,26^ Bulk magnetization measurements of the single crystals were performed in a magnetic property measurement system (MPMS3-SQUID, Quantum Design) over a temperature range of 2–300 K and under applied magnetic fields of up to 7 T. X-ray diffraction analysis was performed at room temperature using a SuperNova X-ray diffractometer, with the crystal mounted on a four-circle Kappa goniometer. For reference in the XPS experiments, a 7.4 nm-thick Co film was grown in situ on epitaxial Au(111) film on a mica substrate using e-beam evaporation, followed by annealing at 300 °C for 15 min^39^

### X-ray Magnetic Circular Dichroism

X-ray absorption spectroscopy (XAS) and X-ray magnetic circular dichroism (XMCD) measurements were performed at the HECTOR endstation of BL29-BOREAS at the ALBA synchrotron in Barcelona, Spain, at a base temperature of 2 K with an applied magnetic field of up to 6 T along the beam direction. Complementary XAS and XMCD measurements were performed at the high-field magnet endstation of the ID32 beamline at the European Synchrotron Radiation Facility (ESRF) in Grenoble, France, at a base temperature of 5 K and an applied magnetic field of up to 9 T along the beam direction. The Co_3–*x*_Ni_*x*_Sn_2_S_2_ single crystals were cleaved in situ along the (001) plane to provide unoxidized, clean surfaces for the measurements. XAS was carried out in total-electron yield mode at the Co and Ni L_2,3_ edges. The XMCD spectra, which reveal the respective element-specific magnetic properties, were obtained by calculating the difference between the two absorption spectra acquired with opposite helicities of the incident X-ray beam, oriented parallel and antiparallel to the applied magnetic field.

The spin and orbital magnetic moments for Co are determined through the sum-rule analysis.^35^ By integrating the XMCD spectra over the L_3_ and L_2,3_ edges, the values *p* and *q*, respectively, are obtained. Next, by integrating the sum spectrum of XAS over the L_2,3_ edge to obtain the normalization factor *r* accounting for the number of holes in the 3d shell, *n*_h_ = 10 – *n*_*d*_, the orbital and spin moments per Co atom are obtained as *m*_l_ = −(4/3)*qn*_h_/*r* and *m*_s_ = −(6*p* – 4*q*)*n*_h_/*r*.^36^

### Soft and Hard X-ray Photoemission Spectroscopy

Soft and hard X-ray photoemission spectroscopy (XPS) measurements were carried out in the EH2 endstation of beamline I09 at the Diamond Light Source,^49^ UK, under ultrahigh vacuum conditions with a base pressure below 2 × 10^–10^ mbar. Soft X-rays (1300–1500 eV) and hard X-rays (6600 eV) were aligned to the same spot on the sample to ensure consistency in the measurements. The spectra were recorded by a Scienta EW4000 electron analyzer. The energy resolutions achieved were better than 200 meV for soft X-rays and 300 meV for hard X-rays. The angle between the hard (soft) X-ray beam and the analyzer is 93°(87°), and the sample surface normal was oriented to be 45° from the analyzer. All single crystals were cleaved in situ along the (001) plane to achieve clean surfaces, and kept at temperatures of either 90 or 30 K, depending on the cooling method used (liquid nitrogen or helium). The photon energies were precisely calibrated using the Fermi edge of a gold reference and cross-validated against the Fermi edge of the cleaved single crystals to ensure accuracy in the spectral data.

### X-ray Photoemission Electron Microscopy

X-ray photoemission electron microscopy (XPEEM) measurements were performed at the SPEEM station of UE49-PGMa beamline at BESSY II,^50^ in Berlin, Germany, at a base temperature of 37 K. The single crystals were cleaved in situ along the (001) plane to expose fresh surfaces, and subsequently cooled down to the base temperature under zero magnetic field. XPEEM contrast was achieved via XMCD, i.e., by calculating the difference image from XAS images taken with opposite circular polarizations at the Co L_3_ edge.
